# 3T BOLD MRI with low intrascan variability and high reproducibility of limb oxygenation measurements

**DOI:** 10.1186/1532-429X-14-S1-P282

**Published:** 2012-02-01

**Authors:** Erik Hedstrom, Ashish S Patel, Tobias Voigt, Bijan Modarai, Tobias Schaeffter, Alberto Smith, Eike Nagel

**Affiliations:** 1 Division of Imaging Sciences and Biomedical Engineering, Kings College London, London, UK; 2 Academic Department of Surgery, St Thomas’ Hospital, Kings College London, London, UK; 3 BHF Centre of Research Excellence and NIHR Biomedical Research Centre at Guy's and St Thomas' NHS Foundation Trusts and King's College London, London, UK; 4 Philips Research, Clinical Research Europe, London, UK

## Background

Current imaging methods, including BOLD MR imaging, cannot reliably quantify muscle oxygenation in patients with limb ischaemia. The BOLD response is complex and measurements have so far been performed in relatively low-resolution EPI-readout images where it may be difficult to exclude oxygenation changes from partial volume effects. The reproducibility of BOLD imaging has been low and consequently its usefulness as a clinical test has been limited. We propose a high-resolution BOLD sequence whereby edge artefacts and vessels may be excluded from measurements, analysed using a Maximum Likelihood Estimate with Rician noise correction, where noise is read in k-space, for a precise and unbiased estimation of T2* from magnitude images. We test the hypothesis that this gives high reproducibility at baseline, and during and after transient ischaemia.

## Methods

The lower limbs of eight volunteers without limb disease (median age 66±3yrs) were imaged twice for reproducibility at time intervals between 1 and 190 days at 3T with a 32-channel coil (Philips Achieva, Best, NL). During each scan transient ischaemia was induced by application of a compression cuff around the thigh inflated to suprasystolic pressure for 5mins. The multi-echo multi-shot GRE BOLD images (TR 66ms, TE1 4.6 ms, ΔTE 4.6 ms, 14 echoes, 1535 Hz/px, FA 20°, FOV 300×150mm, matrix 256×128) were acquired every 2s at baseline and during the dynamic phases of ischaemic response. Data was analysed for early (20s) and late (280s) during cuffing, and for early (20s) and late (315s) after cuff deflation, and interscan reproducibility assessed. Intrascan variability was also determined in an uncuffed population (n=12; median age 67±2yrs). Regions of interest were drawn around the anterior (ant) and lateral (lat) muscle compartments and around the gastrocnemius (gc) and the soleus (sol) muscles, masking out edge artefacts and vessels.

## Results

**Figure 1:**
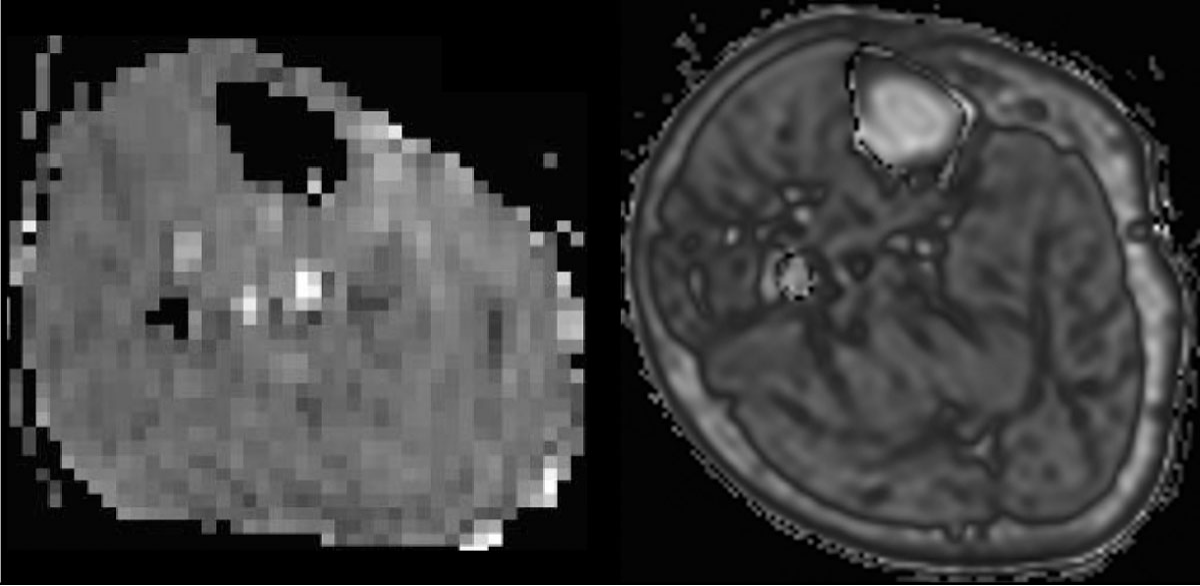
T2* maps by standard EPI BOLD (left) compared with the proposed non-EPI GRE BOLD sequence (right).

**Figure 2:**
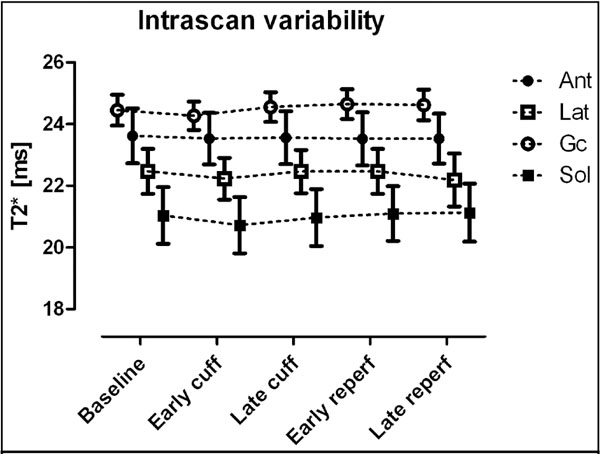
Intrascan variability for the four compartments in an un-cuffed population. Error bars indicate standard error of the mean. Ant=anterior compartment; Lat=lateral compartment; Gc=gastrocnemius; Sol=soleus.

Image resolution was superior to standard EPI BOLD (Figure 1). Intrascan variability for each muscle group was: ant<0.1±0.5ms, lat<0.3±0.9ms, gc<0.2±0.5ms, and sol<0.1±0.6ms (all p values between intrascan time points for each muscle group=NS), Figure 2. Interscan reproducibility for baseline was: ant<1.3±1.9ms, lat<1.7±1.2ms, gc<1.0±1.0ms, and sol<0.6±2.5ms; for minimum T2* during ischaemia ant<0.8±0.2ms, lat<0.9±1.9ms, gc<0.7±1.0ms, and sol<2.1±1.6ms; for maximum T2* early after cuff deflation ant: <0.2±1.0ms, lat: <3.0±0.3ms, gc: <1.0±1.1ms, and sol: <0.9±0.7ms; and for T2* late after cuff deflation ant<0.8±1.2ms, lat<2.4±1.5ms, gc<0.9±0.3ms, and sol<0.9±0.6ms (all p=NS). Compared with baseline, the minimum and maximum T2* values were -19±7% and 1±7%, respectively.

## Conclusions

The sequence and analysis proposed shows low intrascan variability and high interscan reproducibility for measurement of T2*. The technique is therefore well suited for serial assessment of limb muscle oxygenation.

## Funding

British Heart Foundation. The National Institute for Health Research (NIHR) Biomedical Research Centre (BRC) at Guy’s and St Thomas’ NHS Foundation Trust and King’s College London. Lund University Medical Faculty (Stiftelsen Regementsläkaren dr Hartelii stipendiestiftelse) and Lund University Hospital, Sweden. The Foundation BLANCEFLOR Boncompagni-Ludovisi, née Bildt, Sweden. The Swedish societies of Medicine, Cardiology, and Radiology. Covidien.

